# Lifestyle-Related Factors and Atopy in Seven Danish Population-Based Studies from Different Time Periods

**DOI:** 10.1371/journal.pone.0137406

**Published:** 2015-09-15

**Authors:** Tea Skaaby, Lise Lotte Nystrup Husemoen, Betina Heinsbæk Thuesen, Torben Jørgensen, Allan Linneberg

**Affiliations:** 1 Research Centre for Prevention and Health, Capital Region of Denmark, Glostrup, Denmark; 2 Faculty of Health and Medical Sciences, University of Copenhagen, Copenhagen, Denmark; 3 Faculty of Medicine, Alborg University, Alborg, Denmark; 4 Department of Clinical Experimental Research, Glostrup University Hospital, Glostrup, Denmark; Université Paris Descartes, FRANCE

## Abstract

**Background:**

The prevalence of allergic respiratory disease tends to increase in populations that adopt the so-called Westernized lifestyle. We investigated the association between atopy and several possible lifestyle-related factors in seven Danish population-based studies.

**Methods:**

A total of 20048 persons participated in the seven studies. We used logistic regression to analyse the associations between possible determinants and atopy defined as serum specific IgE or skin prick test positivity against inhalant allergens. Associations were expressed as odds ratios (ORs) with 95% confidence intervals (95% CIs). In addition, individual participant data meta-analyses were performed.

**Results:**

Atopy was significantly associated with younger age (OR per 1 year increase in age: 0.97; 95% CI: 0.97, 0.98); male sex (OR for males versus females: 1.34; 95% CI: 1.24, 1.45), heavy drinking (OR for heavy drinkers versus light drinkers: 1.15; 95% CI: 1.04, 1.27), never smoking (OR for current versus never smokers: 0.73; 95% CI: 0.67, 0.80), and higher educational level (OR for educated versus uneducated: 1.27; 95% CI: 1.15, 1.41). Atopy was not associated with blood pressure, serum total cholesterol, physical activity or body mass except in women only, where we found a positive association (OR for obese vs. normal weight: 1.18; 95% CI: 1.00, 1.39) with p_trend_ = 0.032.

**Conclusions:**

Of interest for preventive purposes, we found that atopy was associated with some of the reversible lifestyle-related factors that characterize a Westernized lifestyle.

## Introduction

The prevalence of allergic respiratory disease such as allergic rhinitis and allergic asthma has increased over recent decades in countries living a Westernized, urbanized, and affluent lifestyle [1,2]. However, the causes of this increase are largely unknown [3]. The rapid rise in the prevalence of allergic respiratory disease is not likely to be explained by changes in genetic factors but rather by changes in environmental factors [4]. Although the environment clearly influences the risk of allergic respiratory disease, there has been increasing attention to lifestyle factors as possible determinants of allergic respiratory disease. For example, there is evidence that obesity is associated with the development of asthma [5–9], but the association with allergic sensitization and the possible mechanisms are not clear [10–12]. Although cholesterol is believed to promote allergic inflammation in rodents [13], few adult studies have addressed this association [4,14]. Likewise, alcohol is a strong immune modulating factor and alcohol consumption has been found to increase serum total IgE levels, but the role of the raised total IgE levels in IgE-mediated allergic reactions and IgE-mediated allergic respiratory disease is not clear [15–20]. Smoking has been suggested to increase the risk of allergic symptoms by increased inflammation in the airways, but it is still unresolved whether smoking confers an increased or a decreased risk of allergy [21].

Many of the previous studies on lifestyle-related factors of allergic respiratory disease have mainly been performed in children or adolescents, have not used objective measures of allergic respiratory disease, or been single-study analyses with limited power to detect associations. In seven Danish population-based studies of adults from different time periods, we investigated the association between several potential lifestyle-related factors of atopy, i.e. serum total cholesterol, systolic blood pressure, smoking habits, alcohol intake, leisure time physical activity, body mass index, age, gender and education, and atopy, defined as specific IgE or skin prick test positivity against inhalant allergens that are both well-accepted objective biomarkers of allergic respiratory disease.

## Materials and Methods

### Ethics statement

All the included studies were approved by the Ethics Committee of Copenhagen and the Danish Data Protection Agency. We followed the recommendations of the Declaration of Helsinki, and each participant gave informed written consent.

### Study populations

We used the seven population based studies: Monica1, Inter99, Health2006, the 1936-cohort, Allergy98, Health2008 and Health2010. Participants were recruited from the Danish Central Personal Register as random samples of the background population living in the Western part of the Copenhagen Region [22]. All studies included comprehensive questionnaire and interview data as well as clinical and biochemical data [22].

The Monica1 study took place from 1982 to 1984. A total of 4,807 persons aged 30, 40, 50 and 60 years old were invited, 3,785 persons participated, and thus the participation rate was 79% [22].

The Health2010 study was conducted between 2010 and 2012 where 3732 persons between 18 and 69 years of age were invited. A total of 1522 persons participated which meant a 40.5% participation rate [23].

A total of 12,934 persons between 30 and 60 years of age were invited to the Inter99 study that took place between 1999 and 2001. The study was a population-based intervention study (CT00289237, ClinicalTrials.gov) that examined the effects of lifestyle intervention on the incidence of cardiovascular disease [24,25]. Data from the baseline examination before intervention was used in the present analyses. A total of 6,784 persons participated, and thus the participation rate was 52.5% [25]. The Health2006 study was conducted between 2006 and 2008. A total of 7,931 persons from 18 to 69 years of age were invited, and 3,471 (participation rate 43.8%) persons were examined [26].

The Health2008 study was conducted between 2008 and 2009 [22,27,28]. A total of 2218 persons 30–60 years of age were invited. Pregnant women, persons with known diabetes, chronic obstructive pulmonary disease, cardiovascular disease, hypertension, a history of blood clots, or unable to participate in physical activities such as climbing stairs were excluded from the study. Thus, a total of 795 participated (participation rate 36%).

The Copenhagen Allergy study that included two groups of persons began in 1990. The first group was randomly selected from the general population, and the other group included persons with allergic respiratory symptoms that were chosen by a screening questionnaire from a random sample of the general population. In the present study, we used data from the follow-up study in 1997–1998 (referred to as ‘Allergy98’). In the Allergy98 study, 1,966 persons aged 15–77 years with Danish nationality were invited, and 1,216 (participation rate 61.9%) participated [29].

The 1936-cohort study took place in 1976–1977, where 1,200 randomly selected persons born in 1936 (40 years of age at the time) were invited to a health examination that focused on cardiovascular risk. A total of 1,052 persons were examined, and the participation rate was 87.7% [30,31].

A total of 20048 persons participated in the seven studies. We excluded 2654 persons that had no data on specific IgE measurements or skin prick test and 249 persons that had participated in one of the former studies. Thus, we included a total of 17145 persons from the seven studies (Monica1: 3481, 1936-cohort: 989, Allergy98: 1172, Inter99: 5961, Health2006: 3246, Health2008: 792, Health2010: 1504). Please also see the S1 Fig and S1 Table.

### Covariates

The questionnaires provided data on the covariates education (no education beyond basic [basic education includes primary and lower secondary education for nine or ten years], education including students); physical activity during leisure time (sedentary, light, or moderate/vigorous); alcohol consumption (abstinent: 0, light drinkers >0–7, moderate drinkers >7–14, or heavy drinkers >14 drinks per week); and smoking habits (never smokers, former smokers, current smokers). Year of birth was divided into decades (nineteen twenties, nineteen thirties, nineteen fifties, nineteen sixties or nineteen seventies and above) except for the last group that contained persons born in the nineteen seventies, nineteen eighties, and nineteen nineties.

Height and weight were measured, and body mass index (BMI) was calculated as weight divided by height squared, expressed in kg/m^2^ and classified as (underweight: <18.5, normal weight: 18.5-<25, overweight: 25-<30, and obese: ≥30 kg/m^2^). Serum total cholesterol in mmol/l and systolic blood pressures in mmHg were used as continuous variables.

The number of missing values was: education (N_missing_ = 298); physical activity during leisure time (N_missing_ = 205); smoking habits (N_missing_ = 332); alcohol consumption (N_missing_ = 735); BMI (N_missing_ = 7); total cholesterol (N_missing_ = 13); and systolic blood pressure (N_missing_ = 38) giving a total of 1317 persons (7.7%) with a missing value in at least one of the covariates. S2 Table shows the baseline characteristics of the participants with no missing variables.

### Atopy

Atopy was defined by determination of serum specific IgE or skin prick test positivity to inhalant allergens as described in previous studies [32–35]. In the 1936-cohort and the Monica1 study, serum specific IgE positivity was tested by the ADVIA Centaur Allergy Screen assay (Bayer HealthCare Diagnostics division, Tarrytown, N.Y., USA) [36]. This is a multi-allergen assay to detect specific serum IgE antibodies to 19 different common inhalant allergens. Atopy was defined as a positive result according to the manufacturer’s instructions.

In the Health2006, Health2008 and Allergy98 studies, we used the ADVIA Centaur sIgE assay (Bayer Corporation, New York, NY) to test serum specific IgE to mite (Dermatophagoides [D.] pteronyssinus), grass, cat, and birch (In the Allergy98 study in addition dog and mugwort) [37]. In the Inter99 study, serum samples were analyzed for specific IgE to mite (D. pteronyssinus), grass, cat, and birch by the IMMULITE 2000 Allergy Immunoassay System [38]. In the Allergy98, the Inter99, the Health2006, and the Health2008 study, the specific IgE analysis was positive if the measurement was ≥0.35 kU/l. Specific IgE positivity, atopy, was defined as one or more positive tests for specific IgE against the tested allergens.

In the Health2010 study, skin prick testing (SPT) was performed by the Soluprick SQ® (ALK Abelló, Hørsholm, Denmark) system. The procedure was performed using lancets and a standard panel of aeroallergens comprising birch, grass (Phleum pratense) mugwort, horse, cat, dog, two house dust mites (Dermatophagoides pteronyssinis and D. farinae), and two moulds (Cladosporium herbarum and Alternaria alternata). A negative control and a positive control (10 mg/mL histamine) were also included. The mean wheal diameter was calculated as the average of the widest diameter and the perpendicular bisector. A positive SPT was defined as a mean wheal diameter ≥3 mm. Atopy was defined as a positive reaction to at least one of ten allergens.

### Statistical analyses

The analyses were performed with SAS, version 9.4 (SAS Institute Inc. Cary, NC USA). P-values were two-sided and p-values <0.05 defined as statistically significant. Table 1 shows the baseline characteristics of the participants according to study population and is expressed as mean (standard deviation, SD) or % (number).

**Table 1:** Characteristics of the study populations.

	Mean (SD) or % (n)						
	1936-cohort	Monica1	Allergy98	Inter99	Health2006	Health2008	Health2010
Age (years)	40.4 (0.4)	45.0 (11.1)	40.0 (15.1)	46.1 (7.9)	49.0 (13.1)	46.8 (8.2)	48.8 (13.9)
Systolic BP (mmHg)	120.7 (14.0)	123.6 (16.9)	128.9 (17.9)	130.2 (17.4)	130.3 (17.9)	121.6 (15.2)	129.0 (18.2)
S-cholesterol (mmol/l)	6.3 (1.2)	6.1 (1.2)	5.8 (1.3)	5.5 (1.1)	5.3 (1.1)	5.3 (1.0)	5.3 (1.0)
**Gender**							
Male	46.7 (462)	50.6 (1761)	45.7 (535)	49.1 (2930)	45.2 (1467)	43.6 (345)	44.1 (664)
Female	53.3 (527)	49.4 (1720)	54.3 (637)	50.9 (3031)	54.8 (1779)	56.4 (447)	55.9 (840)
**Alcohol** (drinks/week)							
0	20.3 (201)	14.5 (504)	19.2 (225)	9.7 (550)	6.6 (194)	7.0 (52)	10.0 (139)
>0–7	43.1 (426)	47.8 (1664)	48.2 (564)	45.1 (2562)	48.2 (1422)	55.1 (411)	50.0 (698)
>7–14	17.2 (170)	18.3 (635)	19.4 (227)	21.7 (1235)	22.7 (670)	21.3 (159)	20.9 (291)
>14	19.4 (192)	19.4 (675)	13.2 (154)	23.5 (1337)	22.5 (662)	16.6 (124)	19.1 (267)
**Education**							
Basic	28.5 (282)	29.9 (1041)	28.8 (336)	16.8 (962)	13.8 (440)	8.4 (66)	15.4 (229)
Beyond basic	71.5 (706)	70.1 (2440)	71.2 (832)	83.2 (4777)	86.2 (2756)	91.6 (717)	84.6 (1263)
**BMI** (kg/m^2^)							
<18.5	3.0 (30)	2.1 (73)	1.3 (15)	1.0 (60)	1.7 (56)	1.0 (8)	0.6 (10)
18.5-<25	65.2 (645)	58.0 (2018)	49.4 (579)	42.1 (2507)	46.6 (1511)	48.4 (383)	45.4 (683)
25-<30	26.0 (257)	30.8 (1071)	33.4 (392)	39.7 (2367)	35.5 (1151)	34.7 (275)	38.0 (571)
≥30	5.8 (57)	9.1 (318)	15.9 (186)	17.2 (1023)	16.2 (526)	15.9 (126)	16.0 (240)
**Physical activity**							
Sedentary	34.7 (343)	28.3 (985)	26.0 (303)	21.6 (1257)	18.5 (595)	14.8 (117)	18.0 (268)
Light	51.0 (505)	51.4 (1787)	50.3 (587)	61.5 (3581)	60.4 (1938)	58.2 (460)	57.2 (851)
Moderate/vigorous	14.3 (141)	20.3 (705)	23.7 (277)	16.9 (981)	21.1 (676)	27.0 (214)	24.8 (369)
**Smoking habits**							
Current smokers	54.0 (534)	53.8 (1874)	39.9 (463)	36.8 (2093)	22.8 (733)	18.3 (145)	17.8 (266)
Former smokers	13.2 (131)	16.1 (561)	17.8 (207)	26.5 (1505)	32.2 (1035)	33.4 (264)	35.5 (529)
Never smokers	32.8 (324)	30.1 (1046)	42.3 (492)	36.7 (2087)	45.0 (1446)	48.3 (382)	46.7 (696)
**Atopy**							
Non-atopics	85.1 (842)	82.9 (2887)	62.7 (735)	65.5 (3903)	76.7 (2488)	72.7 (576)	70.3 (1057)
Atopics*	14.9 (147)	17.1 (594)	37.3 (437)	34.5 (2058)	23.3 (758)	27.3 (216)	29.7 (447)

Abbreviations: BMI, body mass index; BP, blood pressure; SD, standard deviation.

* Serum specific IgE or skin prick test positivity to inhalant allergens.

Multivariate logistic regression analyses were used to model the cross-sectional associations between different covariates and atopy. In model 1, we adjusted for gender, age, and study population (with an indicator of the study population that the participant belonged to). In model 2, we further adjusted for birth year (except when using age as exposure), education, physical activity, smoking habits, alcohol intake, BMI, systolic pressure and serum total cholesterol. We used complete case analysis, i.e. persons with missing values in one or more of the covariates were excluded from the regression analyses.

In additional analyses, we only included the six studies that defined atopy according to serum specific IgE positivity. We also stratified by gender the analyses regarding BMI and atopy.

**Fig 1:**
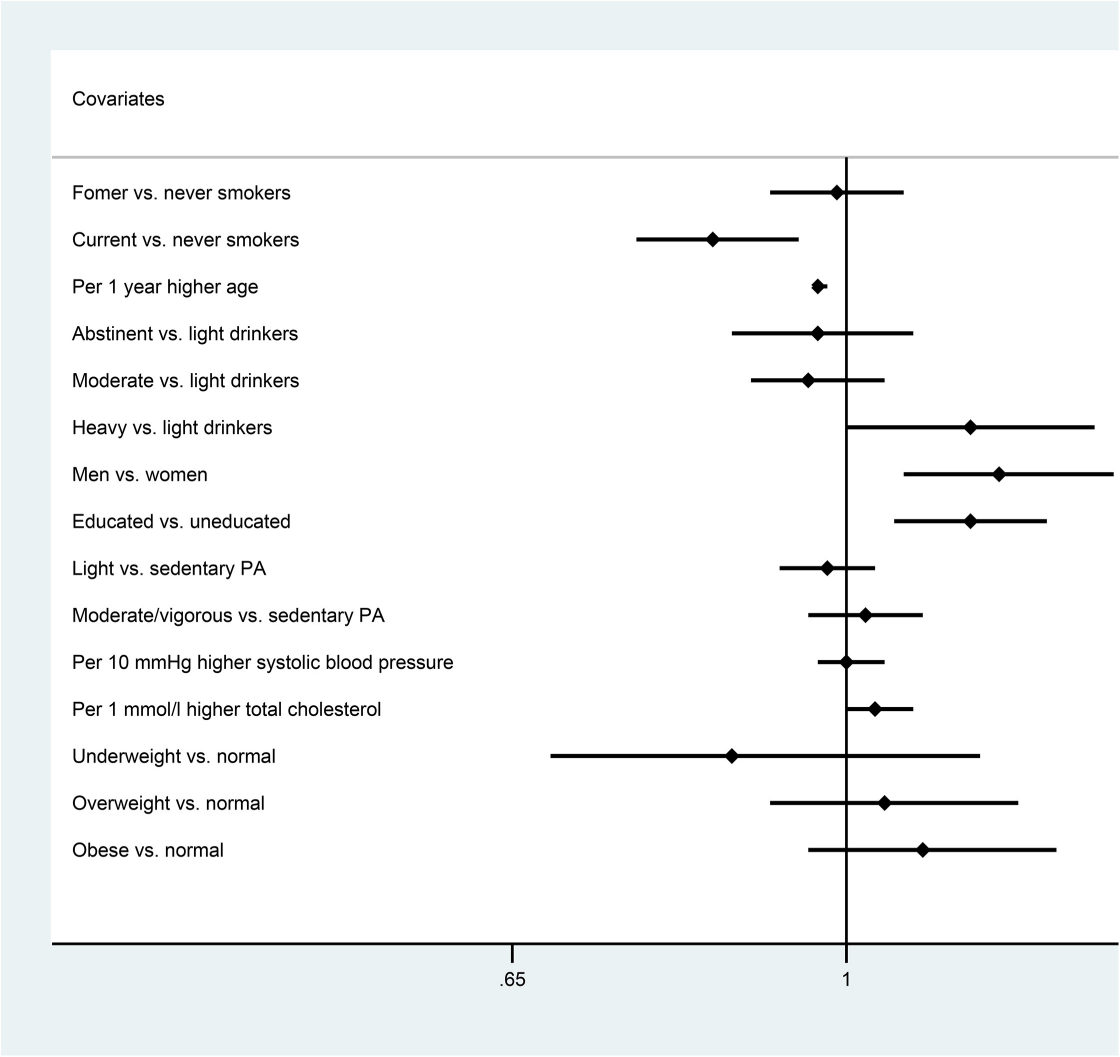
Overview of the results of meta-analyses of the study-specific estimates of the associations of atopy with possible lifestyle-related factors. Abbreviations: OR, odds ratio; CI, confidence interval; vs., versus.

We used Stata, version 12.1 (StataCorp LP, College Station, Texas, USA), to perform meta-analyses of the fully adjusted study specific estimates obtained from logistic regression models for each study separately. This is an alternative to the analyses of the pooled studies, mainly to account for the higher correlation within studies than between studies and to assess the heterogeneity across the studies. We combined the OR estimates with the inverse variance method in random rather than the fixed effects models because of heterogeneity across studies for some of the analyses, detected by the I^2^-test. An overview of the results from meta-analyses is shown in Fig 1.

The specific meta-analyses are shown in S2–S10 Fig. Due to the narrow age span in the 1936-cohort study, we excluded it from the meta-analysis of age and atopy (S5 Fig).

## Results

Participants’ birth years were distributed over the following decades (nineteen twenties: 5.1% (n = 874), nineteen thirties: 13.8% (n = 2368), nineteen forties: 24.6% (n = 4219), nineteen fifties: 29.3% (n = 5023), nineteen sixties: 18.1% (n = 3096); or nineteen seventies and above: 9.1% (n = 1565).

The mean age of the study participants varied between approximately 40 years (allergy98) to approximately 50 years (Health2006) (Table 1). In all but one study, Monica1, more females participated. The number of uneducated, sedentary participants and serum cholesterol levels were highest in the old studies and lower in the younger ones as opposed to BMI and the number of never smokers and atopics that were lowest in the old studies and somewhat higher in the youngest studies. Systolic blood pressure and alcohol intake showed no consistent time trends across the studies.

In the adjusted analyses of the merged study data, atopy was significantly associated with young age, male sex, high intake of alcohol, never smoking, and education beyond basic school, but not with systolic blood pressure or serum cholesterol (Table 2).

**Table 2:** The associations of atopy with possible factors of atopy (n = 15828).

	OR (95% CI) of atopy compared to reference group/per increase	
	Model 1*	Model 2**
**Age** (years)		
Per 1 year older	0.98 (0.97, 0.98)	0.97 (0.97, 0.98)
P-value	<0.0001	<0.0001
**Systolic blood pressure** (mmHg)		
Per 10 mmHg higher systolic blood pressure	1.02 (0.99, 1.04)	1.00 (0.97, 1.02)
P-value	P = 0.224	0.881
**Serum cholesterol** (mmol/l)		
Per 1 mmol/l higher s-cholesterol	1.03 (1.00, 1.07)	1.03 (1.00, 1.07)
P-value	0.092	0.077
**Gender**		
Male	1.41 (1.31, 1.51)	1.34 (1.24, 1.45)
Female	1 (reference)	1 (reference)
P-value	<0.0001	<0.0001
**BMI** (kg/m^2^)		
<18.5	0.85 (0.61, 1.18)	0.89 (0.64, 1.23)
18.5-<25	1 (reference)	1 (reference)
25-<30	1.06 (0.98, 1.15)	1.05 (0.97, 1.15)
≥30	1.07 (0.96, 1.19)	1.06 (0.94, 1.19)
P-value***	0.085	0.176
**Physical activity**		
Sedentary	1 (reference)	1 (reference)
Light	1.01 (0.92, 1.10)	0.98 (0.89, 1.07)
Moderate/vigorous	1.06 (0.95, 1.18)	1.02 (0.91, 1.14)
P-value***	0.318	0.790
**Alcohol** (drinks/week)		
0	0.96 (0.85, 1.09)	0.99 (0.87, 1.12)
>0–7	1 (reference)	1 (reference)
>7–14	1.00 (0.90, 1.10)	1.00 (0.91, 1.11)
>14	1.10 (1.00, 1.22)	1.15 (1.04, 1.27)
P-value***	0.050	0.017
**Smoking habits**		
Current smokers	0.73 (0.67, 0.80), p<0.0001	0.73 (0.67, 0.80), p<0.0001
Former smokers	0.82 (0.75, 0.90), p = 0.296	0.81 (0.74, 0.89), p = 0.194
Never smokers	1 (reference)	1 (reference)
**Education**		
Basic	1 (reference)	1 (reference)
Beyond basic	1.31 (1.19, 1.44)	1.27 (1.15, 1.41)
P-value	<0.0001	<0.0001

* Adjusted for gender, age, and study population.

** Further adjusted for birth year (except for age), education, physical activity, smoking habits, alcohol intake, BMI, systolic blood pressure and serum total cholesterol.

*** P-value for trend.

Abbreviations: OR, odds ratio; BMI, body mass index; CI, confidence interval.

The additional analyses including only the six studies using serum specific IgE positivity for the definition of atopy (the Health2010 study was excluded) showed similar results (data not shown). The meta-analyses of the study-specific estimates were quite similar to results of the pooled data analyses which shows that differences between the studies did not seriously bias our results (Fig 1 and S2–S10 Figs).

BMI was not associated with atopy in men only (OR for underweight vs. normal weight: 0.83; 95% CI: 0.40, 1.70; OR for overweight vs. normal weight: OR = 1.03; 95% CI: 0.92, 1.16; and OR for obese vs. normal weight: 0.96; 95% CI: 0.81, 1.13). The p-value for trend among men was p_trend_ = 0.891. However, BMI was significantly associated with atopy in women (OR for underweight vs. normal weight: OR = 0.91; 95% CI: 0.63, 1.32; OR for overweight vs. normal weight: 1.07; 95% CI: 0.95, 1.22; and OR for obese vs. normal weight: 1.18; 95% CI: 1.00, 1.39). The p-value for trend among women was p_trend_ = 0.032.

## Discussion

In analyses including more than 20,000 persons, we found that atopy was significantly associated with younger age, male sex, heavy drinking, not smoking, and educational level, but not with blood pressure, serum cholesterol, physical activity or body mass index. The large sample size allowed us to estimate the independent effects of these factors with a reasonably high precision. Data come from a pool of seven large population-based studies conducted on Danish adults since the early 1980's up to recent years, and the use of objective factors of atopy and standardized methods in general enabled the exploration of disease prevalence and risk factors over time [1,39,40].

The observed positive trend between time and prevalence of atopy is well-known and in line with several previous reports using parts of the data used in the present analyses [1,39,40]. The increasing trends of atopy support questionnaire/interview surveys on symptoms/diagnoses performed in Denmark in this period. The associations of atopy with male sex [18,41], higher education [41], and young age [18,31,42] are in line with several previous studies [42]. A possible mechanism underlying the association with age could be that the immune response in general decreases with increasing age rendering older persons less susceptible to allergic reactions and novel sensitisation. An alternative explanation could be that younger generations may have been exposed to novel environmental risk factors in early life that have changed their susceptibility to allergy. This so-called cohort effect is believed to have mainly affected generations born from the 1960s and onwards [40].

Although smoking is a known risk factor for asthma, its association with development of allergic sensitization is controversial. In line with the observed lower prevalence of atopy among current smokers compared to never smokers in the present study, Linneberg et al found that smoking was associated with a lower prevalence [18] and incidence [43] of IgE-mediated sensitization to inhalant allergens. Wüthrich et al also found smoking to be negatively associated with atopy in the previously mentioned cross-sectional Swiss study of 8344 persons [42]. On the other hand, Gergen et al found no association between smoking and atopy [44] and—although not entirely comparable to our results—, a recent meta-analysis by Saulyte et al. found no associations between active smoking and *allergic rhinitis* [21]. One possible explanation for a negative association between smoking and atopy may be that smokers who develop allergies tend to quit smoking, i.e., an example of reverse causation, although this is inconsistent with studies reporting that the risk of atopy among former smokers is intermediate of that of current and never smokers [18]. The association may also reflect an immunosuppressive effect of smoking [43]. We found statistically significant heterogeneity between studies in the analyses of current smoking and odds ratio of atopy (S4 Fig). Considering the individual study results, the 1936 cohort seems to be the cause of heterogeneity, since it is the only study with a positive association with atopy. Therefore, we consider this finding incidental.

The effect of alcohol consumption on sensitisation is also not clear. In 2003, Linneberg et al found that alcohol consumption was positively associated with serum total IgE but not with IgE sensitisation [19], and in 2010, Linneberg et al found that alcohol consumption was associated with prevalent but not incident aeroallergen sensitisation [31]. Also, Assing et al found no association between alcohol consumption and skin prick test positivity among 1668 Danish students [15]. The observed positive association between alcohol intake and atopy in the present study is, however, in line with several other studies [16,18,20]. The possible mechanisms by which alcohol consumption could affect allergic sensitization are not fully understood but may include a direct effect on the B-cells or the alcohol-induced increased permeability in the gut lumen [17]. There is also evidence that high intake of alcohol increases IgE sensitization to cross-reactive carbohydrate determinants that may interfere with allergy testing [31,45].

Previous studies examining the association between obesity and atopy in adults have shown conflicting results [9]. There are both studies supporting a positive association [46–48] and studies against such an association [49–58]. The positive association in the present study between BMI and atopy among women in particular, is in line with several studies that include mainly children or adolescents [56–58]. The mechanisms by which obesity might be related to atopy—and possibly in women only—are not clear but include a possible influence of sex hormones on the development and expression of atopy and atopic disorders [59–61] or the fact that for a given BMI, women have a higher percentage of body fat than men [9]. However, given the fact that overweight and obesity are in increase, it is important to address any possible effect of overweight and obesity in the pathogenesis of atopy in future studies.

In addition, theoretically both serum lipids and blood pressure could mediate a possible effect of obesity on atopy since obesity has a detrimental effect on both, and both are related to some form of inflammation [62]. However, we found no association between either systolic blood pressure or serum total cholesterol and atopy. The lack of association between serum total cholesterol and atopy is somewhat in line with a nested case-control study by Schäfer et al who found no association between serum total cholesterol and allergic sensitization in adjusted analyses [4]. However, Fessler et al found that the odds ratio of atopy defined as serum specific IgE positivity against allergens was OR = 1.17 (95% CI: 1.00, 1.38) per two standard deviation increase in total cholesterol which is comparable to ORs of serum cholesterol for myocardial infarction [14]. The possible underlying mechanism may be an effect of serum lipids on pro-atopic Th2 immunity and allergic inflammation [13,14].

The strengths of our study include the large population-based samples and the inclusion of multiple studies. Also a strength, is the use of objective factors of atopy, i.e. serum specific IgE positivity and skin prick test positivity against inhalant allergens, which may be more reliable than self-reported diagnoses and symptoms. Although different methods were employed for assessment of atopy, our previous comparison of e.g. the ADVIA Centaur Allergy Screen assay (used in the 1936-cohort and the Monica1 studies) against skin prick test reactivity (used in the Health2010 study) showed good agreement in our background population [36]. We have also compared the ADVIA Centaur sIgE assay (used in the Health2006, Health2008 and Allergy98 studies) against skin prick test positivity showing good agreement [37]. In general, previous studies have also shown good agreement in the different ways of measuring serum specific IgE against inhalant allergens [63,64].

One limitation is that the questionnaires used for collection of data on symptoms and diagnoses of diseases in the various studies differed markedly over time period and some studies lacked questions on allergic respiratory disease. Hence, we could not perform meaningful analyses for these outcomes across populations.

In conclusion, in this large cross-sectional study we confirmed that atopy, objectively assessed by IgE or skin prick test sensitization to inhalant allergens, is associated with sex and age and in addition with several lifestyle-related factors. Although environment is clearly important in the aetiology allergic respiratory disease, lifestyle factors could also contribute to the allergy epidemic.

## Supporting Information

S1 Fig Overview of the participants. (TIF)

S2 Fig Random effects meta-analyses of the study-specific associations between alcohol intake and atopy. Abbreviations: a98, Allergy98; h06, Health2006; h08, Health2008; h10, Health2010; i99, Inter99; k36, 1936-cohort; m1, Monica1; OR, odds ratio; CI, confidence interval.
(TIF)

S3 Fig Random effects meta-analyses of the study-specific associations between gender and atopy. Abbreviations: a98, Allergy98; h06, Health2006; h08, Health2008; h10, Health2010; i99, Inter99; k36, 1936-cohort; m1, Monica1; OR, odds ratio; CI, confidence interval.
(TIF)

S4 Fig Random effects meta-analyses of the study-specific associations between smoking status and atopy. Abbreviations: a98, Allergy98; h06, Health2006; h08, Health2008; h10, Health2010; i99, Inter99; k36, 1936-cohort; m1, Monica1; OR, odds ratio; CI, confidence interval.
(TIF)

S5 Fig Random effects meta-analyses of the study-specific associations between age and atopy. Abbreviations: a98, Allergy98; h06, Health2006; h08, Health2008; h10, Health2010; i99, Inter99; k36, m1, Monica1; OR, odds ratio; CI, confidence interval.
(TIF)

S6 Fig Random effects meta-analyses of the study-specific associations between educational level and atopy. Abbreviations: a98, Allergy98; h06, Health2006; h08, Health2008; h10, Health2010; i99, Inter99; k36, 1936-cohort; m1, Monica1; OR, odds ratio; CI, confidence interval.
(TIF)

S7 Fig Random effects meta-analyses of the study-specific associations between leisure time physical activity and atopy. Abbreviations: a98, Allergy98; h06, Health2006; h08, Health2008; h10, Health2010; i99, Inter99; k36, 1936-cohort; m1, Monica1; OR, odds ratio; CI, confidence interval.
(TIF)

S8 Fig Random effects meta-analyses of the study-specific associations between systolic blood pressure and atopy. Abbreviations: a98, Allergy98; h06, Health2006; h08, Health2008; h10, Health2010; i99, Inter99; k36, 1936-cohort; m1, Monica1; OR, odds ratio; CI, confidence interval.
(TIF)

S9 Fig Random effects meta-analyses of the study-specific associations between serum total cholesterol and atopy. Abbreviations: a98, Allergy98; h06, Health2006; h08, Health2008; h10, Health2010; i99, Inter99; k36, 1936-cohort; m1, Monica1; OR, odds ratio; CI, confidence interval.
(TIF)

S10 Fig Random effects meta-analyses of the study-specific associations between body mass index and atopy. Abbreviations: a98, Allergy98; h06, Health2006; h08, Health2008; h10, Health2010; i99, Inter99; k36, 1936-cohort; m1, Monica1; OR, odds ratio; CI, confidence interval.
(TIF)

S1 Table Description of the included studies. Study participants were drawn as a random samples of the general population living in a defined area of the Western part of Copenhagen.
(DOCX)

S2 Table Characteristics of the study populations (persons with no missing values, N = 15,828). (DOCX)
