# Correction: Lifestyle-Related Factors and Atopy in Seven Danish Population-Based Studies from Different Time Periods

**DOI:** 10.1371/journal.pone.0141403

**Published:** 2015-10-20

**Authors:** The PLOS ONE Staff

Fig 1, “Overview of the results of meta-analyses of the study-specific estimates of the associations of atopy with possible lifestyle-related factors”, appears incorrectly due to an error in the typesetting process. The publisher apologizes for this error. Please see the correct Fig 1 here.

**Fig 1:**
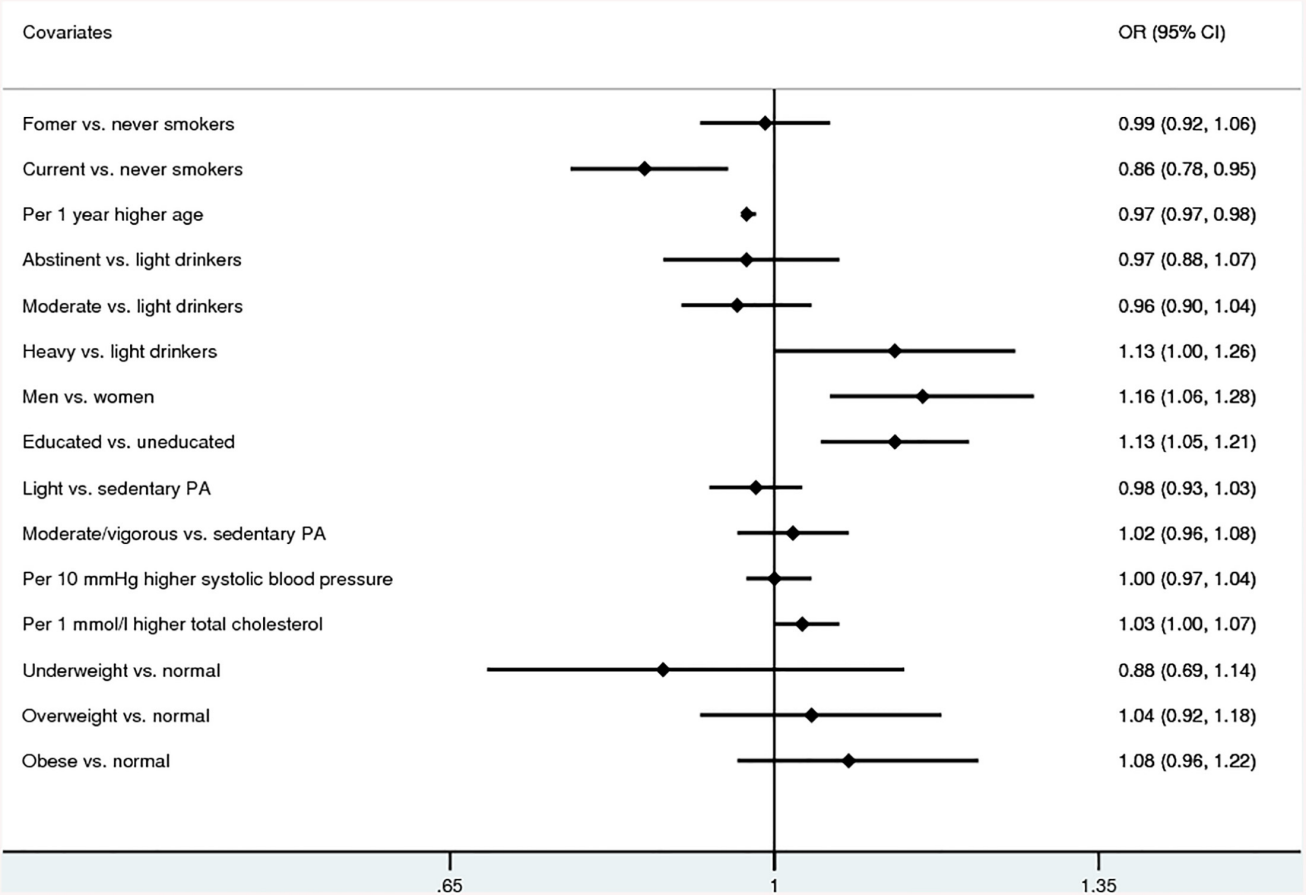
Overview of the results of meta-analyses of the study-specific estimates of the associations of atopy with possible lifestyle-related factors. Abbreviations: OR, odds ratio; CI, confidence interval; vs., versus.
