# Supplementary figures and images for: Lifestyle-Related Factors and Atopy in Seven Danish Population-Based Studies from Different Time Periods
Source: PLoS One. 2015 Sep 15;10(9):e0137406. doi: 10.1371/journal.pone.0137406 (PMC4570778; doi:10.1371/journal.pone.0137406)

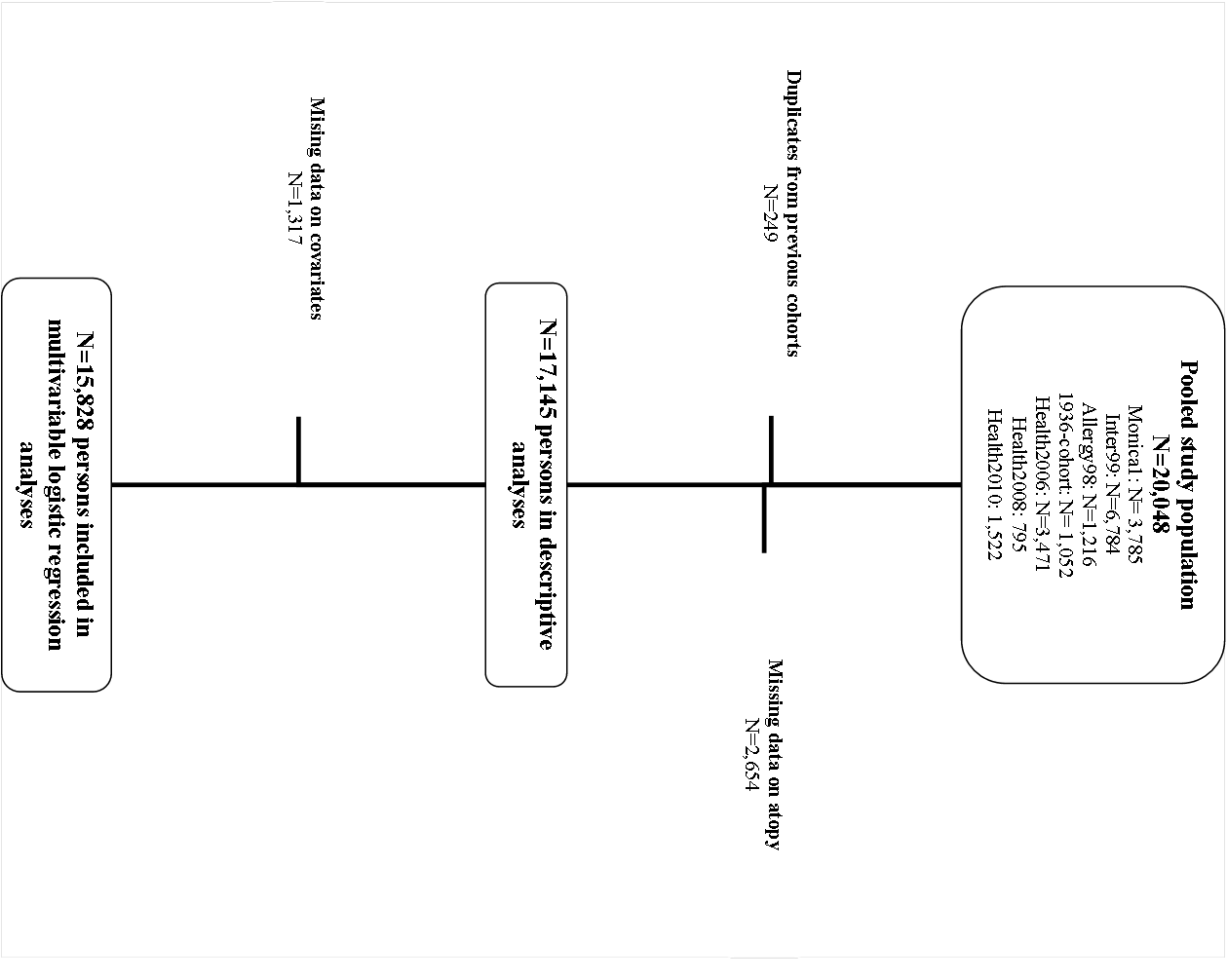

Supplement: S1 Fig — (TIF) [file pone.0137406.s001.tif]

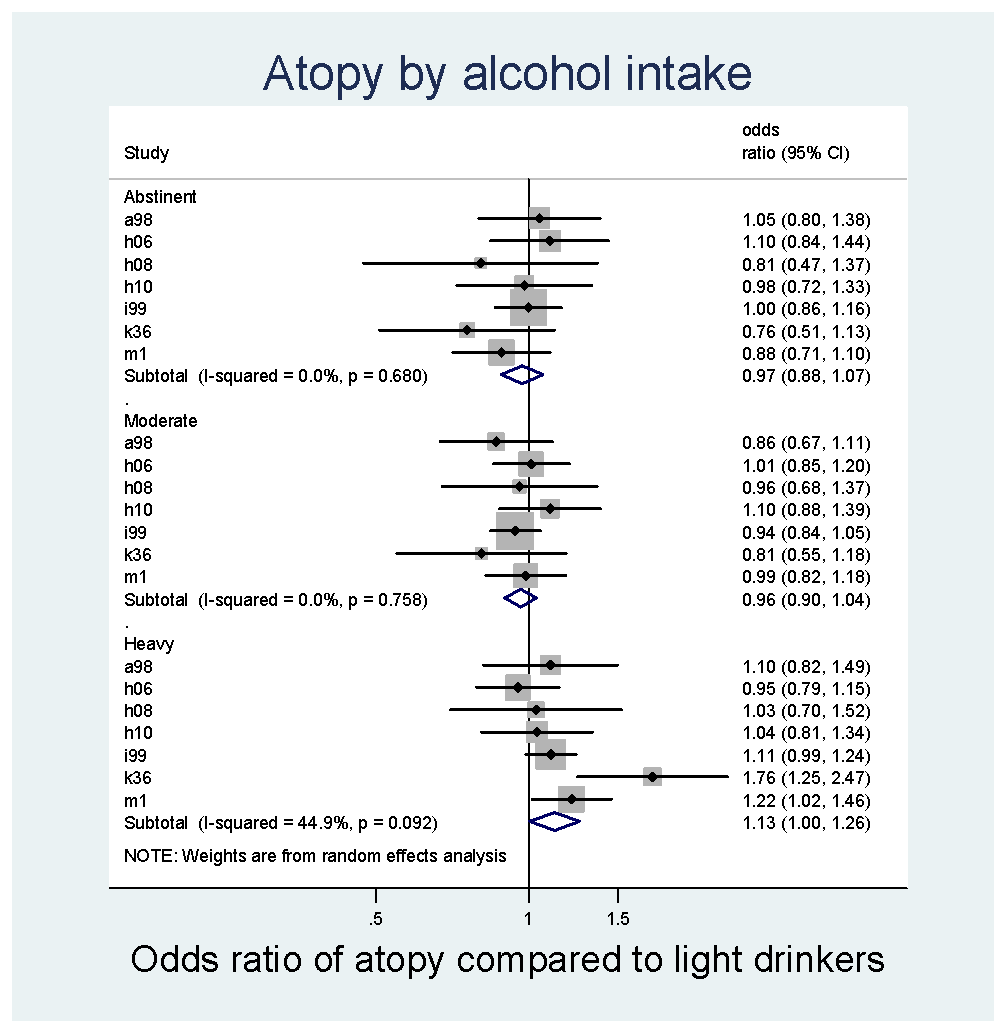

Supplement: S2 Fig — Abbreviations: a98, Allergy98; h06, Health2006; h08, Health2008; h10, Health2010; i99, Inter99; k36, 1936-cohort; m1, Monica1; OR, odds ratio; CI, confidence interval. (TIF) [file pone.0137406.s002.tif]

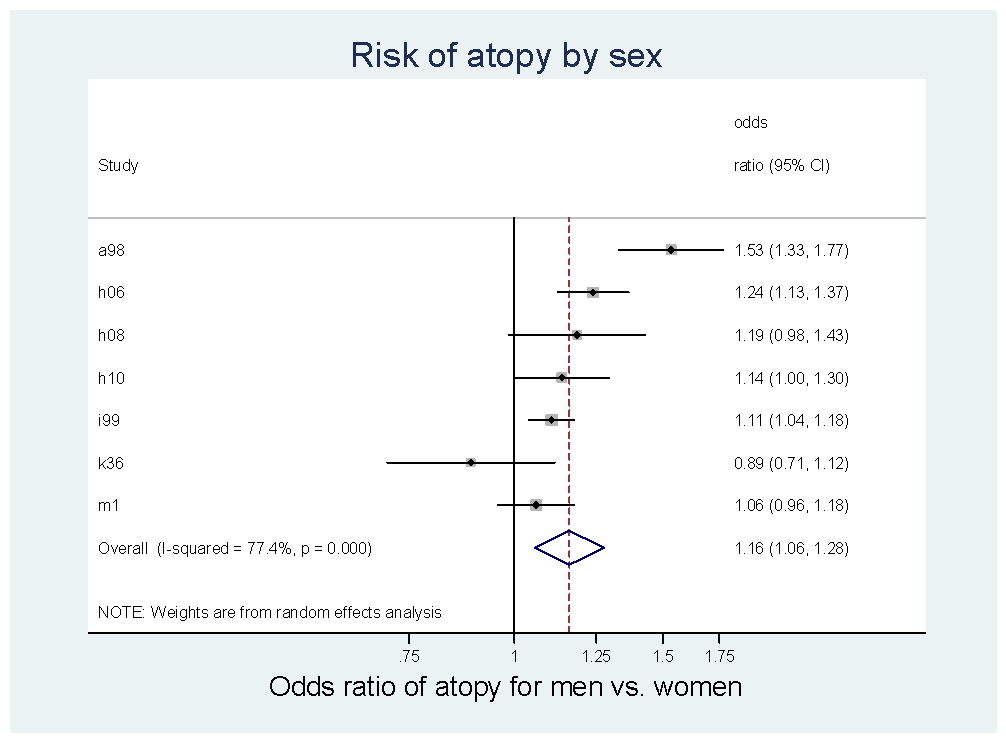

Supplement: S3 Fig — Abbreviations: a98, Allergy98; h06, Health2006; h08, Health2008; h10, Health2010; i99, Inter99; k36, 1936-cohort; m1, Monica1; OR, odds ratio; CI, confidence interval. (TIF) [file pone.0137406.s003.tif]

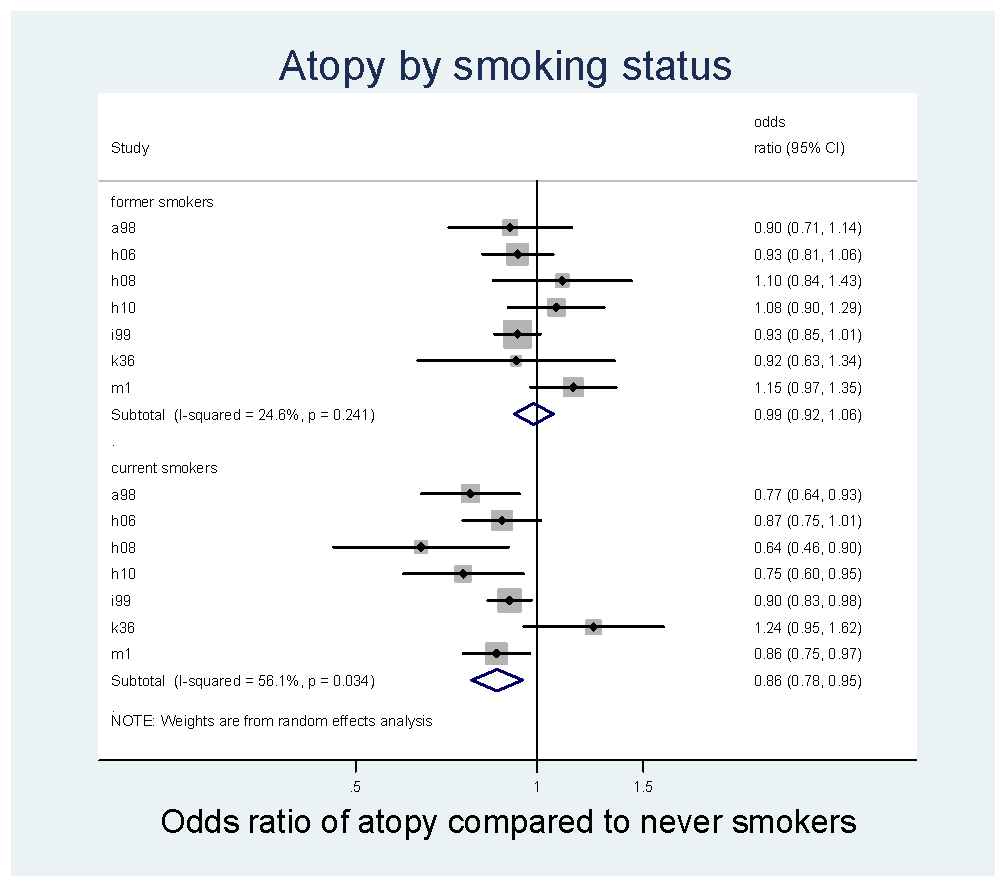

Supplement: S4 Fig — Abbreviations: a98, Allergy98; h06, Health2006; h08, Health2008; h10, Health2010; i99, Inter99; k36, 1936-cohort; m1, Monica1; OR, odds ratio; CI, confidence interval. (TIF) [file pone.0137406.s004.tif]

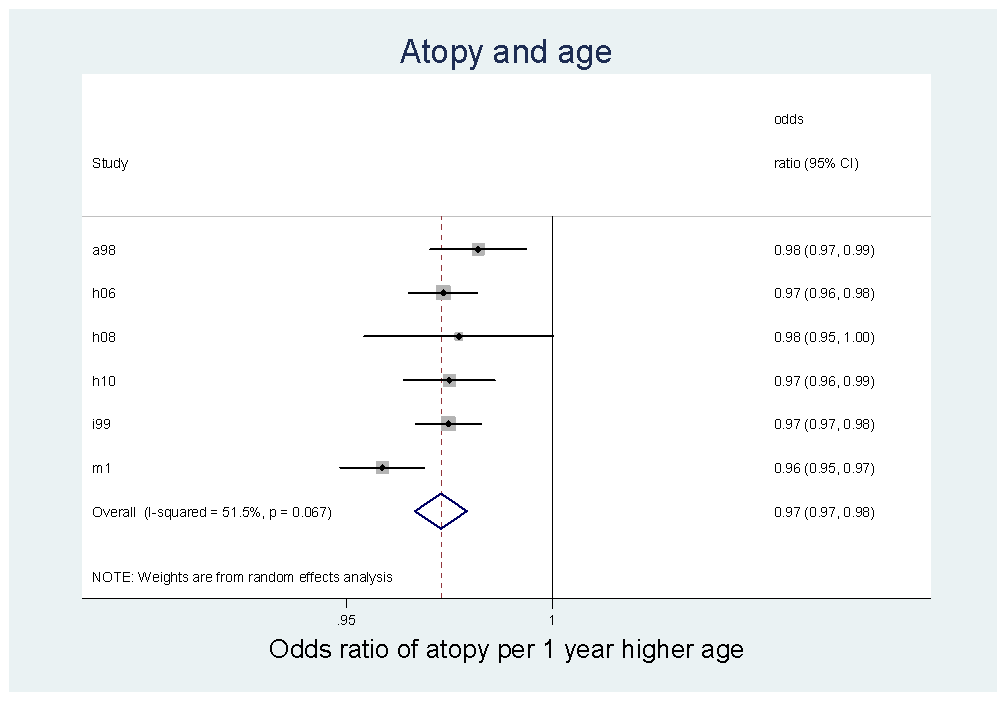

Supplement: S5 Fig — Abbreviations: a98, Allergy98; h06, Health2006; h08, Health2008; h10, Health2010; i99, Inter99; k36, m1, Monica1; OR, odds ratio; CI, confidence interval. (TIF) [file pone.0137406.s005.tif]

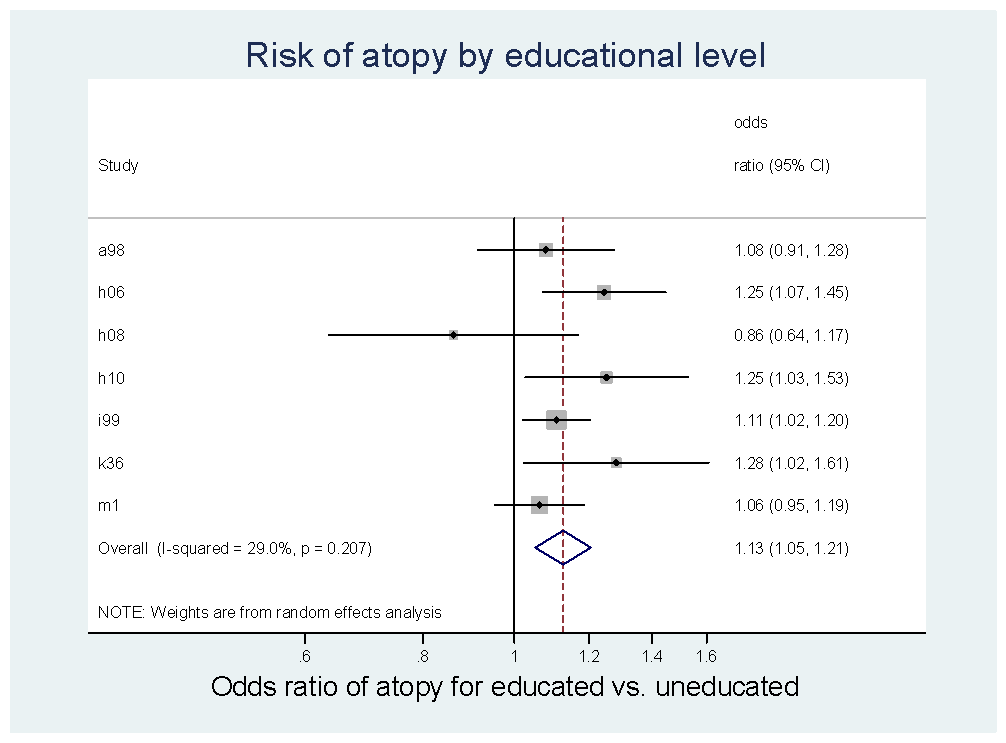

Supplement: S6 Fig — Abbreviations: a98, Allergy98; h06, Health2006; h08, Health2008; h10, Health2010; i99, Inter99; k36, 1936-cohort; m1, Monica1; OR, odds ratio; CI, confidence interval. (TIF) [file pone.0137406.s006.tif]

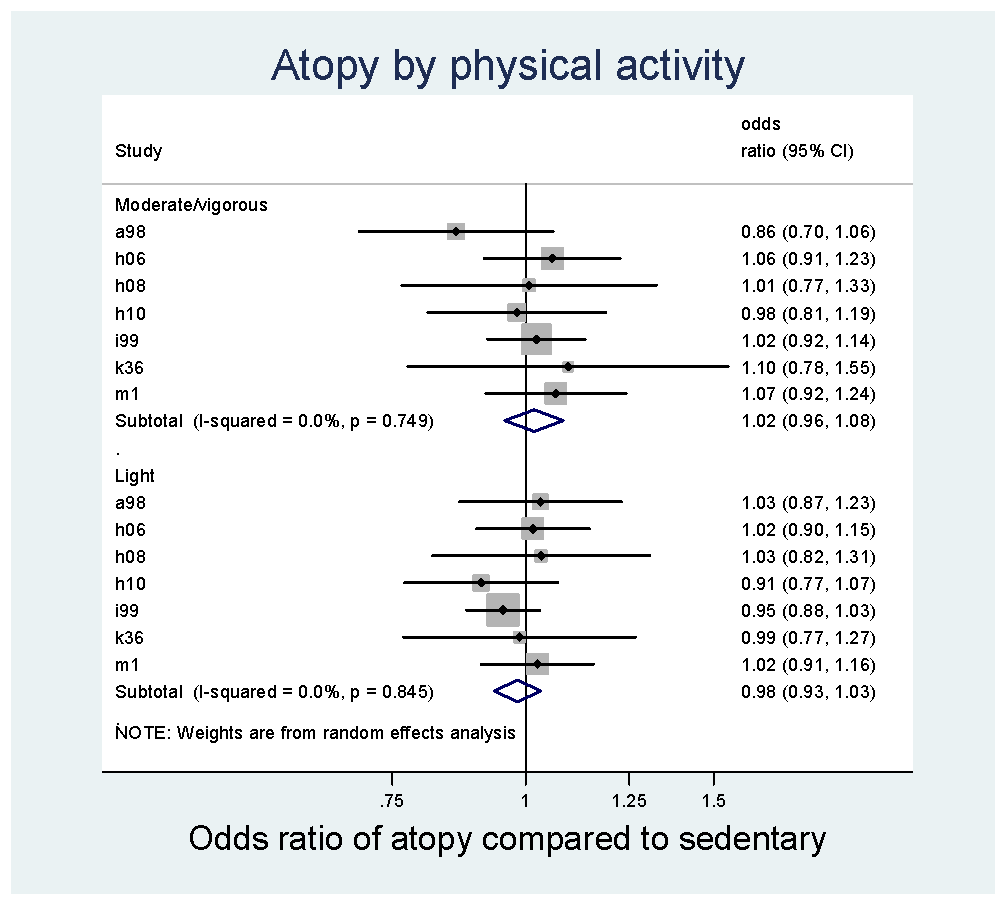

Supplement: S7 Fig — Abbreviations: a98, Allergy98; h06, Health2006; h08, Health2008; h10, Health2010; i99, Inter99; k36, 1936-cohort; m1, Monica1; OR, odds ratio; CI, confidence interval. (TIF) [file pone.0137406.s007.tif]

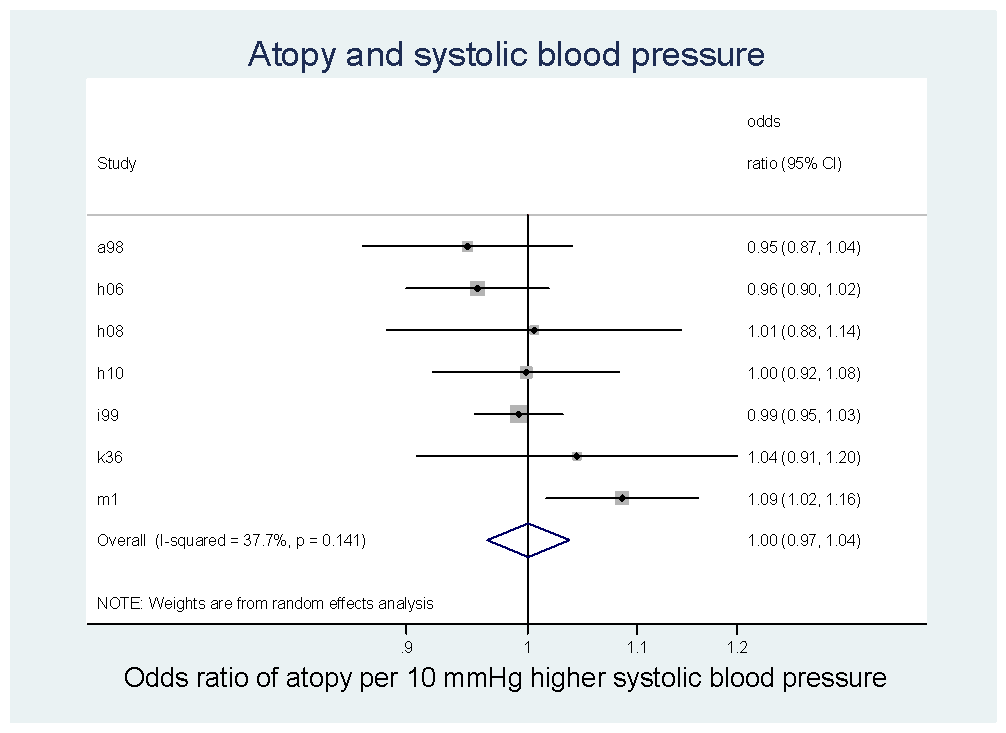

Supplement: S8 Fig — Abbreviations: a98, Allergy98; h06, Health2006; h08, Health2008; h10, Health2010; i99, Inter99; k36, 1936-cohort; m1, Monica1; OR, odds ratio; CI, confidence interval. (TIF) [file pone.0137406.s008.tif]

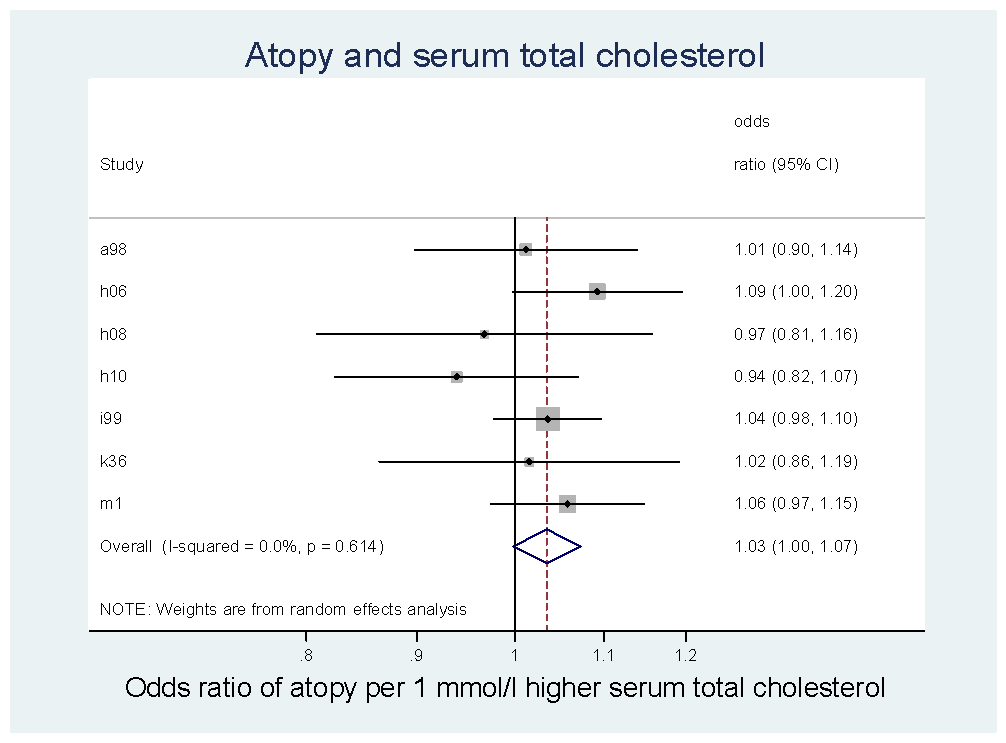

Supplement: S9 Fig — Abbreviations: a98, Allergy98; h06, Health2006; h08, Health2008; h10, Health2010; i99, Inter99; k36, 1936-cohort; m1, Monica1; OR, odds ratio; CI, confidence interval. (TIF) [file pone.0137406.s009.tif]

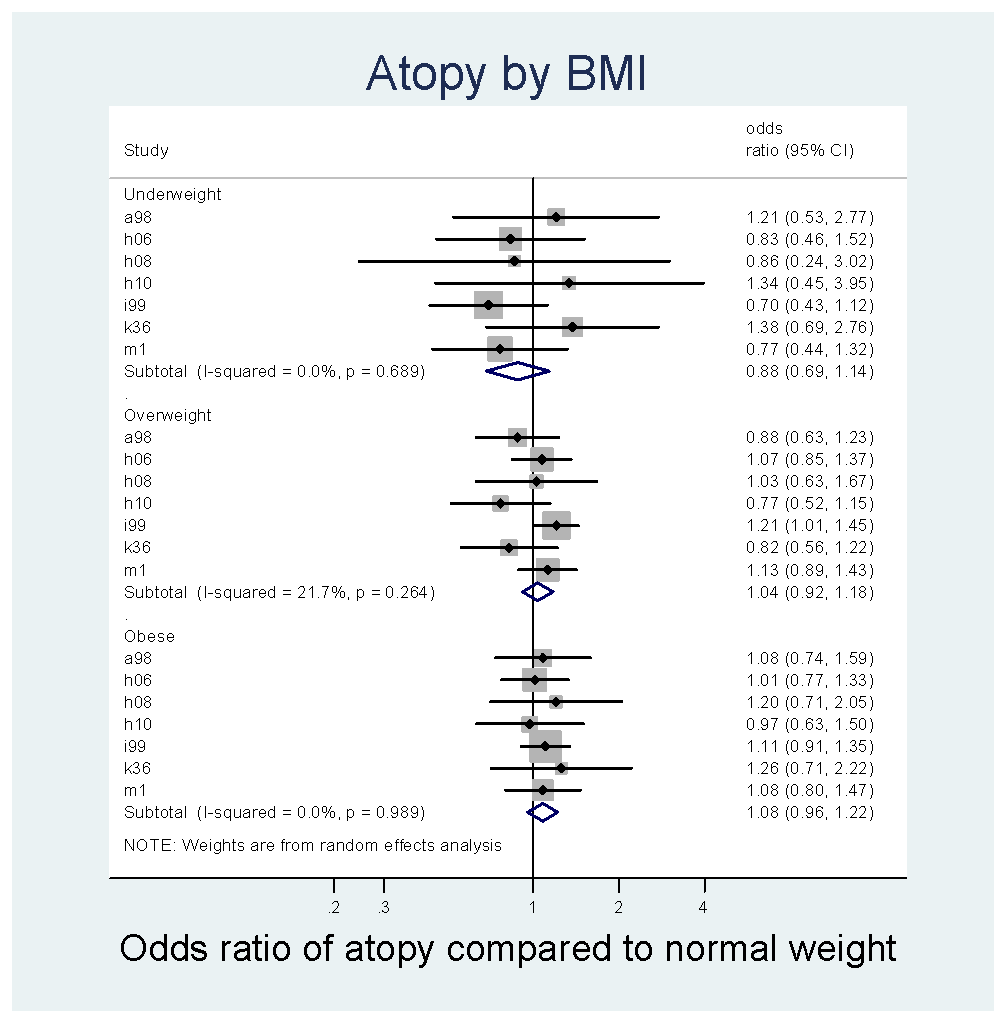

Supplement: S10 Fig — Abbreviations: a98, Allergy98; h06, Health2006; h08, Health2008; h10, Health2010; i99, Inter99; k36, 1936-cohort; m1, Monica1; OR, odds ratio; CI, confidence interval. (TIF) [file pone.0137406.s010.tif]
