# Supplementary material for: Lifestyle-Related Factors and Atopy in Seven Danish Population-Based Studies from Different Time Periods
Source: PLoS One. 2015 Sep 15;10(9):e0137406. doi: 10.1371/journal.pone.0137406 (PMC4570778; doi:10.1371/journal.pone.0137406)
Supplement: S1 Table — Study participants were drawn as a random samples of the general population living in a defined area of the Western part of Copenhagen. (DOCX) [file pone.0137406.s011.docx]

| **Study** | **1936-cohort** | **Monica1** | **Health2006** | **Health2008** | **Health2010** | **Allergy98** | **Inter99** |
| --- | --- | --- | --- | --- | --- | --- | --- |
| **Inclusion criteria** | Random sample of persons born in 1936 | Random age stratified sample of persons aged 30, 40, 50 and 60 years | Random sample of persons aged 18 and 69 years | Random sample of persons aged 30 to 60 years. | Random sample of persons aged 18 and 69 years. | A respiratory symptom stratified random sample of persons aged 18-77 years | An age and sex stratified random sample of persons aged 30, 35, 40, 45, 50, 55 and 60 years |
| **Acronym/ naming** | Named so because all participants were born in 1936. | MONICA is an abbreviation of ‘monitoring cardiovascular  disease’ | Study of health and chronic disease initiated in 2006 | Study of health and chronic disease initiated in 2008 | Study of health and chronic disease initiated in 2010 | Short for Copenhagen Allergy Study 1998. | Multidisciplinary non-pharmacological cardiovascular disease high risk intervention study initiated in 1999 |
| **Year of examination** | 1976-1977 | 1982-1984 | 2006-2008 | 2008-2009 | 2010-2012 | 1997-1998 | 1999-2001 |
| **Study design^*^** | Cohort | Cohort | Cohort | Survey/cohort | Cohort | Cohort | Population-based intervention study |
| **Age, years** | 40 | 30-61 | 18-69 | 30-60 | 18-69 | 15–77 | 30-61 |
| **Sample size** | 1,052 | 3,785 | 3,471 | 795 | 1,522 | 1,216 | 6,784 |

^*^ All data used were cross-sectional.
