# Supplementary material for: Lifestyle-Related Factors and Atopy in Seven Danish Population-Based Studies from Different Time Periods
Source: PLoS One. 2015 Sep 15;10(9):e0137406. doi: 10.1371/journal.pone.0137406 (PMC4570778; doi:10.1371/journal.pone.0137406)
Supplement: S2 Table — (DOCX) [file pone.0137406.s012.docx]

**Table 1. Characteristics of the study populations (N=15,828)**

|  | Mean (SD) or % (n) | | | | | | |
| --- | --- | --- | --- | --- | --- | --- | --- |
|  | **1936-cohort** | **Monica1** | **Allergy98** | **Inter99** | **Health2006** | **Health2008** | **Health2010** |
| Age (years) | 40.4 (0.4) | 45.0 (11.1) | 40.0 (15.2) | 46.0 (7.9) | 49.2 (12.9) | 46.9 (8.2) | 49.1 (13.7) |
| Systolic BP (mmHg) | 120.7 (14.0) | 123.6 (16.9) | 128.8 (17.9) | 130.1 (17.4) | 130.7 (17.8) | 121.7 (15.2) | 129.2 (18.2) |
| S-cholesterol (mmol/l) | 6.1 (1.2) | 6.1 (1.2) | 5.8 (1.3) | 5.5 (1.1) | 5.4 (1.1) | 5.3 (1.0) | 5.3 (1.0) |
| **Gender** |  |  |  |  |  |  |  |
| Male | 46.7 (461) | 50.6 (1758) | 45.8 (511) | 49.6 (2595) | 46.8 (1356) | 44.4 (326) | 45.5 (628) |
| Female | 53.3 (527) | 49.4 (1716) | 54.2 (605) | 50.4 (2642) | 53.2 (1571) | 55.6 (409) | 54.5 (753) |
| **Alcohol** (drinks/week) |  |  |  |  |  |  |  |
| 0 | 20.2 (200) | 14.4 (502) | 18.8 (210) | 9.8 (513) | 6.5 (189) | 7.1 (52) | 10.0 (138) |
| >0-7 | 43.1 (426) | 47.9 (1663) | 48.8 (545) | 45.2 (2369) | 48.1 (1395) | 54.8 (403) | 50.0 (690) |
| >7-14 | 17.2 (170) | 18.3 (634) | 19.4 (217) | 21.5 (1124) | 22.8 (662) | 21.6 (159) | 21.0 (290) |
| >14 | 19.4 (192) | 19.4 (675) | 12.9 (144) | 23.5 (1231) | 22.5 (651) | 16.5 (121) | 19.0 (263) |
| **Education** |  |  |  |  |  |  |  |
| Basic | 28.5 (282) | 29.9 (1037) | 28.9 (323) | 15.6 (818) | 12.4 (358) | 7.9 (58) | 14.4 (199) |
| Beyond basic | 71.5 (706) | 70.1 (2437) | 71.1 (793) | 84.4 (4419) | 87.6 (2539) | 92.1 (677) | 85.6 (1182) |
| **BMI** (kg/m^2^) |  |  |  |  |  |  |  |
| <18.5 | 3.0 (30) | 2.1 (73) | 1.3 (14) | 1.0 (55) | 1.4 (42) | 1.1 (8) | 0.6 (9) |
| 18.5-<25 | 65.3 (645) | 58.0 (2016) | 48.9 (546) | 43.2 (2262) | 47.3 (1370) | 48.8 (359) | 45.2 (624) |
| 25-<30 | 26.0 (257) | 30.8 (1069) | 33.8 (377) | 39.7 (2078) | 35.9 (1040) | 34.4 (253) | 38.5 (531) |
| ≥30 | 5.7 (56) | 9.1 (316) | 16.0 (179) | 16.1 (842) | 15.4 (445) | 15.7 (115) | 15.7 (217) |
| **Physical activity** |  |  |  |  |  |  |  |
| Sedentary | 34.7 (343) | 28.3 (983) | 25.6 (286) | 21.0 (1099) | 17.9 (519) | 14.6 (107) | 17.4 (240) |
| Light | 51.0 (504) | 51.4 (1787) | 50.4 (562) | 62.0 (3245) | 60.1 (1741) | 57.7 (424) | 57.3 (791) |
| Moderate/vigorous | 14.3 (141) | 20.3 (704) | 24.0 (268) | 17.0 (893) | 22.0 (637) | 27.8 (204) | 25.3 (350) |
| **Smoking habits** |  |  |  |  |  |  |  |
| Current smokers | 54.1 (534) | 53.9 (1872) | 39.4 (440) | 36.5 (1912) | 22.4 (650) | 17.8 (131) | 17.3 (239) |
| Former smokers | 13.2 (131) | 16.1 (561) | 18.3 (204) | 26.7 (1396) | 31.8 (921) | 33.7 (248) | 36.2 (500) |
| Never smokers | 32.7 (323) | 30.0 (1041) | 42.3 (472) | 36.8 (1929) | 45.8 (1326) | 48.4 (356) | 46.5 (642) |
| **Atopy** |  |  |  |  |  |  |  |
| Non-atopics | 85.1 (841) | 83.0 (2883) | 62.8 (701) | 65.0 (3402) | 76.2 (2207) | 72.2 (531) | 70.0 (967) |
| Atopics^*^ | 14.9 (147) | 17.0 (591) | 37.2 (415) | 35.0 (1835) | 23.8 (690) | 27.8 (204) | 30.0 (414) |

Abbreviations: BMI, body mass index; BP, blood pressure; SD, standard deviation.

^*^ Serum specific IgE or skin prick test positivity to inhalant allergens.
